# Synthesis of metal anthranilate complexes: catalytic and antipathogenic studies

**DOI:** 10.1186/s13065-022-00817-x

**Published:** 2022-03-27

**Authors:** Muhammad Nawaz, Muhammad Waseem Abbasi, Marium Tariq, John Patrick Graham, Abdul-Rahman Saleh Al-Hagri, Ahmed Awad Elkarim, Muayad Elsiddig Mohamed, Veeranoot Nissapatorn, Muhammad Taha, Soleiman Hisaindee

**Affiliations:** 1 Department of Nano-Medicine Research, Institute for Research and Medical Consultations (IRMC), Imam Abdulrahman Bin Faisal University, P.O. Box 1982, Dammam, 31441 Saudi Arabia; 2 Department of Botany, University of Karachi, Karachi, 75270 Pakistan; 3 M.A.H. Qadri Biological Research Centre, University of Karachi, Karachi, 75270 Pakistan; 4 Department of Life and Physical Sciences, Galway-Mayo Institute of Technology, Galway, Ireland; 5 Chemistry Department, College of Science, United Arab Emirates University, P.O. Box 15551, Al-Ain, United Arab Emirates; 6 School of Allied Health Sciences, World Union of Herbal Drug Discovery (WUHeDD) and Research Excellence Center for Innovation and Health Products (RECIHP), Walailak University, Nakhon Si Thamarrat, 80161 Thailand; 7 Department of Clinical Pharmacy, Institute for Research and Medical Consultations (IRMC), Imam Abdulrahman Bin Faisal University, P.O. Box 1982, Dammam, 31441 Saudi Arabia

**Keywords:** Anthranilic acid, Metal complexes, Catalysis, Environmental pollutants, Biological activities, DFT

## Abstract

**Background:**

Anthranilic acid is an active compound with diverse biological activities such as anti-inflammatory, antineoplastic, anti-malarial and α-glucosidase inhibitory properties. It can also chelate transition metals to form complexes with applications as antipathogens, photoluminescent materials, corrosion inhibitors, and catalysts.

**Results:**

Anthranilic acid complexes (**1–10**) of Zn(II), Bi(III), Ag(I), Fe(II), Co(II), Cu(II), Mn(II), Al, Ni(II), and Cr(III) were synthesized and characterized using thermogravimetric (TGA), elemental analysis, FT-IR, UV–vis spectrometry, mass spectrometry and magnetic susceptibility. The morphology and size of metal complex (**1–10**) particles were determined by scanning electron microscope (SEM) and the surface area was determined by BET analysis. TGA and CHN analysis data indicated that the stoichiometries of complexes were 1:2 metal/ligand except for Ag(I), Al and Bi. Furthermore, DFT study was performed to optimize the structure of selected complexes. The complexes (**1–10**) were evaluated for their catalytic activity in the reduction of 4-nitrophenol (4-NP), antibacterial activity against *S. aureus*, *P. aeroginosa* and *E. coli* as well as their antifungal activity against *F. solani* and *A. niger*. The complexes were also tested against the second-stage juveniles (J_2_) root-knot nematodes.

**Conclusion:**

Co(II) complex **5** and Cu(II) complex **6** showed high catalytic activity for the reduction of 4-NP to 4-aminophenol (4-AP). Ag(I) complex **3** showed the best activity against the pathogens that were tested namely clinically important bacteria *S. aureus*, *P. aeroginosa* and *E. coli,* commercially important fungi *F*. *solani* and *A. niger* and J_2_ root-knot nematodes *M. javanica.*

## Background

Organic-metal complexes are important in medicinal chemistry and are widely used for diagnosis purpose and treatment of different diseases such as arthritis, cancer, anti-microbial, anti-fungal and anti-parasitic agents [1–3]. Transition metals are important in the human body and perform several important functions [4]. Over the past decade, transition metals found an increasing use in drug design and development [4–8].

Industrial waste comprises several hazardous chemicals and materials which seriously affect human health and aquatic animals [9, 10]. Phenolic compounds, organic dyes and pesticides are examples of such industrial wastes [11–13]. Among phenolic compounds, 4-nitrophenol (4-NP) is considered one of the most representative contaminants [14]. 4-Aminophenol (4-AP) is the reduced product of 4-NP and is widely used in the preparation of pharmaceutical drugs [15, 16]. Consequently, numerous efforts have been devoted to get rid of 4-NP by reducing it to 4-AP. Several methods have been employed to resolve this issue such as electrochemical treatment, photocatalytic degradation, microbial degradation and catalytic reduction [17, 18]. It has been observed that reductive catalysis is an effective way to degrade 4-NP [19–21].

Plant pathogens are of economic importance due to their adverse effect on plants and vegetables. They can cause serious damage, affecting quality and productivity of bio products. These pathogens include bacteria, fungi and nematodes. Root-knot nematodes are extensively distributed and are destructive to plants [22]. *Macrophomina* species are root-knot nematodes that cause rotting of stems, roots and pods of several cultured plants [23]. *M. phaseolina* can live in soil and dead plants. Similarly, *Fusarium* species, also cause root and stem diseases among different plant crops [3, 24, 25].

Anthranilic acid has the ability to bind with transition metals due the presence of chelating carboxyl and amino groups and has an important role in medicinal chemistry. It has been reported that anthranilic acid derivatives exhibit anti-bacterial activities [26], and anti-inflammatory [27]. Some derivatives, such as N-phenylanthranilic acid, are employed as pharmacological precursor for the synthesis of anti-neoplastic, anti-inflammatory, anti-malarial [28] and α-glucosidase inhibitors [29].

Herein, we describe the design and synthesis of anthranilic acid metal complexes (**1–10**). The metals used in this study are from groups 6–12 [30], in addition to those of bismuth and aluminum [31], whose complexes possess various biological activities.

Metal complexes were characterized by diverse techniques and their catalytic activity for the reduction of 4-nitropenol was studied. Additionally, we also tested the anthranilic acid metal complexes (**1–10**) against plant pathogens such as bacteria, fungi and nematode.

## Experimental

### Chemicals and methods

The following chemicals were purchased from Sigma Aldrich and their purity is indicated in brackets: Anthranilic acid reagent grade (> 98%), Zinc chloride reagent grade (> 98%), Bismuth(III) nitrate pentahydrate ACS reagent(> 98.0%), Silver nitrate ACS reagent (> 99.0%), Iron(II) sulfate heptahydrate ACS reagent (> 99.0%), Cobalt(II) chloride hexahydrate(> 97%), Copper(II) sulfate pentahydrate ACS reagent(> 98.0%), Manganese(II) chloride tetrahydrate ACS reagent (> 98%), Aluminium sulfate octadecahydrate (> 98%), Zinc nitrate hexahydrate reagent grade (98%), Chromium(III) nitrate nonahydrate (99%). The chemicals were used without further purification.

### Preparation of anthranilic acid metals complexes

Anthranilic acid (0.28 g, 2.0 mmol) was added to 30 mL of deionized water, followed by sodium hydroxide (0.085 g, 2.1 mmol) and the mixture was stirred for half an hour. Then metal salt solutions (0.5 mmol in 5 mL of deionized water) were added to the first solution and additionally stirred for half an hour. The metal salts were in the form of the sulfate, chloride or nitrate (see Table 1). The resulting precipitate was filtered and washed several times with deionized water, dried under vacuum and stored in a desiccator.

**Table 1:** Physical properties of metal complexes (**1–10**) of anthranilic acid (L)

Metal source	Complex number	Molecular Formula	Color of complex	% Yield	Color change on heating (°C)	λ _max_ DMSO (nm)	Magnetic moment (B.M)	IR stretch (cm^−1^)	
								NH:	C=O
ZnCl_2_	**1**	[ZnL_2_]	Beige	83	287 °C to light gray	332.5	0	3297/3129	1600
Bi (NO_3_)_3_.5H_2_O	**2**	[BiL_3_]. H_2_O	Flax yellow	67	279 °C to brown	338.5	0	3305/3124	1614
AgNO_3_	**3**	[Ag_2_L_2_]	Metallic gray	89	187 °C to black	326.0	0	3423/3322	1608
FeSO_4_.7H_2_O	**4**	[FeL_2_]	Lemon green	78	269 °C to dark gray	337.0	5.1	3307/3135	1535
CoCl_2_.6H_2_O	**5**	[CoL_2_]	Pink	42	292 °C to brown	339.0	4.6	3297/3129	1597
CuSO_4_. 5H_2_O	**6**	[CuL_2_]	Green	77	280 °C to black	339.5	1.3	3270/3120	1593
MnCl_2_. 4H_2_O	**7**	[MnL_2_]	Beige	93	272 °C to yellowish	329.0	5.9	3305/3139	1585
Al_2_(SO_4_)_3_. 18H_2_O	**8**	[AlL_3_].3H_2_O	Off white	92	284 °C to brown	339.0	0	3484/3378	1618
Ni (NO_3_)_2_.6H_2_O	**9**	[NiL_2_]	Baby blue	78	290 °C to light brown	337.0	1.31	3305/3124	1614
Cr (NO_3_)_3_.9H_2_O	**10**	[CrL_2_. 4H_2_O]. NO_3_	Purple	79	178 °C to green,	340.0	3.5	–	1616
Anthranilic acid ligand (L)	–	–	–	–	–	340.5	–	3322/3236	1662

### Characterization

FTIR (Perkin-Elmer, Massachusetts, USA) was employed to study the functional groups of the metal complexes of anthranilic acid (**1–10**) and spectra were recorded, as KBr disc, in the range of 400–4000 cm^−1^. Elemental analysis was performed using a Perkin-Elmer instrument. Melting point was obtained from a Stuart (SMP-10) melting point apparatus. UV–Vis spectra of all metal complexes (**1–10**) were recorded in 150 µM DMSO solutions on a UV–visible spectrophotometer (JASCO V-750). Magnetic susceptibility measurements were obtained using a Sherwood Scientific Magnetic Susceptibility Balance. Thermal gravimetric analysis was performed using a Shimadzu Thermogravimetric Analyzer TGA-50 (Shimadzu, Kyoto, Japan) by heating a weighed sample, in air, from room temperature to 600 °C using a ramp of 10 °C/minute. Mass spectra of selected complexes were obtained by direct infusion in a LC/MSD trap 6310 spectrometer (Agilent technologies), equipped with an electrospray ionization source and operated in negative polarity. The mass range was from 210 to 400 Da. Micromeritics ASAP 2020 PLUS (USA) was employed for BET surface area determination, after degassing the samples at 150 °C and surface area was calculated by employing N_2_ adsorption data with range of relative pressure (*P/P*0) 0.05–0.3.

### DFT study

Gaussian 09 [32] was used for all calculations. Iron(II) quintet and Cobalt(II) quartet high-spin complexes were optimized using the UB3LYP [33] functional and 6 − 311 + G(d,p) [34] basis set. Low spin complexes were also modelled but displayed significantly higher total energies. The silver dimer was optimized using B3LYP and the effective core potential Def2TZVP [34] basis set for Ag and 6 − 311 + G(d,p) basis set for all other atoms. Energy minima were confirmed through vibrational frequency calculations. CHELPG [35] charges (charges from electrostatic potentials using a grid-based method) were calculated and used to map the electrostatic potential to the electron density isosurfaces for molecular electrostatic potential surfaces [36].

### Catalytic activity

The catalytic activity of anthranilic acid metal complexes (**1–10**) was investigated towards the reduction of 4-NP as a model compound in the presence of NaBH_4_ (sodium borohydride). To a mixture of 4-NP and sodium borohydride (NaBH_4_) was added metal complexes (**1–10**) separately in quartz cuvette and reaction was incubated at ambient temperature and spectra were recorded at different times (0–70 min) using a UV–Vis spectrophotometer [20, 21].

### Anti-bacterial studies

Bacterial cultures namely *Staphylococcus aureus, Pseudomonas aeroginosa* and *Escherichia coli* were obtained from the culture collection of Department of Microbiology, University of Karachi, Pakistan. These bacterial cultures were further maintained on Nutrient agar. Disc diffusion technique was used to observe antibacterial studies of all compounds against *E. coli*, *S. aureus* and *P. aeroginosa.* For this, lawns of bacterial cells were spread on the surface of Petri plate and disc (6 mm) of tested compounds (with concentrations 500 and 1000 ppm) were placed in two corners of plate. Each disc was impregnated with 50 μL of the test compound. Third corner was occupied with disc containing 50 μL of DMSO as control. Replicates were made for each bacterium, and plates were incubated for 2–4 days at 37 °C, after which, zone of inhibition was measured to the nearest mm around each disc [3, 25].

### Anti-fungal activity

Fungal cultures (*Fusarium solani* and *Aspergillus niger*) were previously isolated from rhizosphere of eggplant (*Solanum melongenosa* L.) grown in Malir district Karachi. Fungal cultures were grown for five days on potato dextrose agar (PDA) before use. Culture discs (6 mm each) were placed in the center of PDA poured plates. Antifungal activities of compounds were tested by disc diffusion method as described above. Data of antifungal activity were recorded at 5–7 days of incubation 37 °C [3, 25].

### Nematicidal activity

Eggplant infected with *Meloidogyne javanica* (root knot nematodes) was collected from the Botany Department, University of Karachi. Egg masses were directly picked from the knots formed on the roots of infected eggplants using a fine needle and placed in sterile water. After hatching, number of juveniles was maintained around 40–60 (1 mL) in each cavity block. Compounds were mixed to 500 and 1000 ppm (prepared in DMSO) in the nematode suspension. Three replicates of each treatment were made and control containing 50 µL of DMSO in sterile water (2 mL) without mixed in compounds. After 24, 48, 72 and 96 h of exposure, dead juveniles were counted. Data of dead nematodes were recorded in mortality % if they did not move when probed with a fine needle [37].

## Results and discussion

### Characterization of anthranilic acid metal complexes

The metal complexes were prepared by mixing a fourfold mole excess of deprotonated anthranilic acid, by prior treatment with sodium hydroxide, with the metal salt aqueous medium. The complexation is fast, and the solid products are collected by filtration, washed and dried. When heated, most of the products showed marked changes in physical appearance without melting (Table 1), due to loss of water and organic material as confirmed by TGA analysis.

**Fig. 1:**
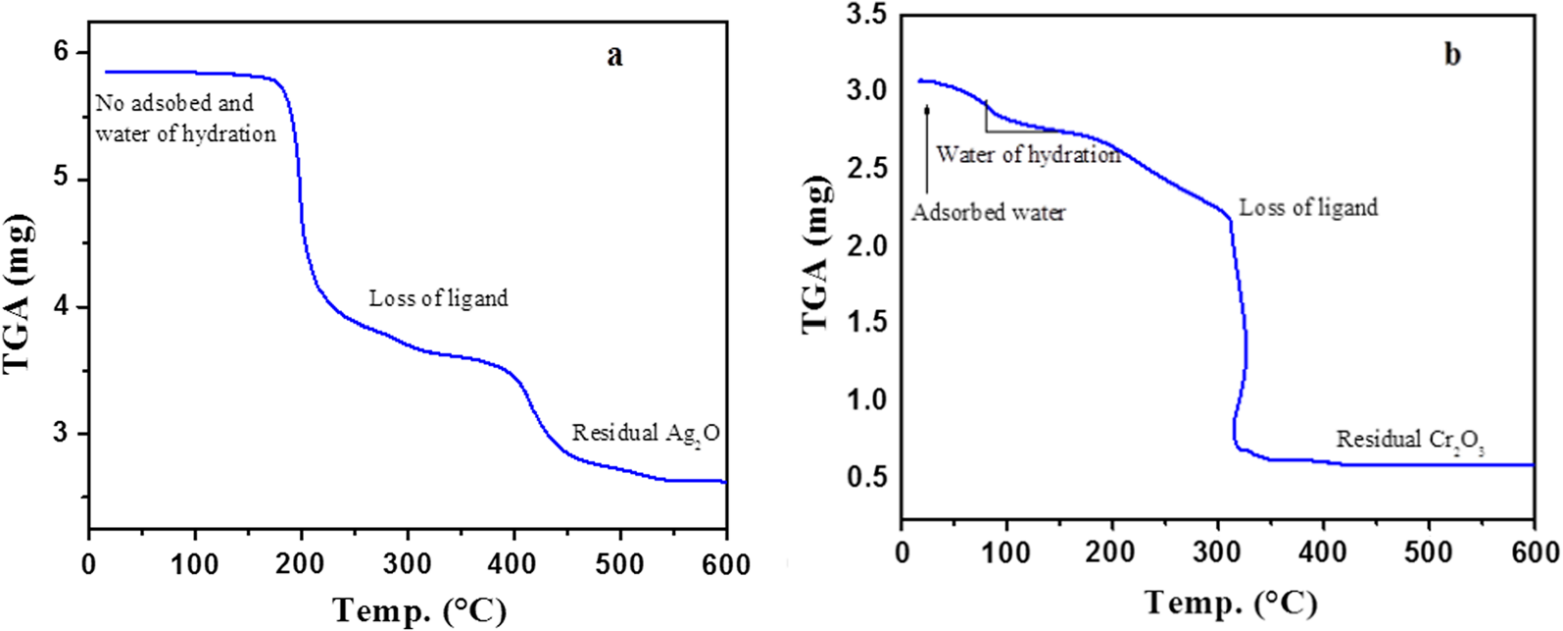
TGA thermograms of anthranilic acid metal complexes of **a** Ag (I) and **b** Cr (III) complexes

The complexes were characterized by TGA to obtain essential information about the presence/absence of water of hydration, which can be obtained by heating the samples above 105 °C. Typical thermogram is shown in Fig. 1a.

The anthranilic complex of Ag (I) **3** shows that the sample does not contain any adsorbed and chemically bound water molecules (water of hydration) as no mass loss was observed at around 105 °C. All the complexes were found to be anhydrous except for Bi (III), Al and Cr (III). A typical thermogram of the hydrated complex is shown for Cr (III) complex **10** (Fig. 1b). Complex **10** contained both types of water molecules. The thermograms were also used to determine the formula of the complexes using a method we reported earlier [3]. The results are shown in Table 2. To confirm the identity of the complexes, we also run the elemental analysis of the complexes (Table 2). The elemental analysis of all the complexes were within acceptable range of error, except for the % N for complex **10**. With the exception of AlL_3_.3H_2_O, all the other complexes have been reported [38] with either anthranilic acid or its derivatives.

**Table 2:** TGA and elemental results of metal complexes (**1–10**)

Complex	Formula		Formula weight	Calculated				Experimental		
				%M	%C	%H	%N	%C	%H	%N
**1**	[ZnL_2_]	ZnC_14_H_12_N_2_O_4_	337.6	38.4	49.8	3.55	8.29	49.7	3.66	7.69
**2**	[BiL_3_]. H_2_O	BiC_21_H_20_N_3_O_6_	635.0	49.6	39.7	3.15	7.55	39.0	2.82	5.96
**3**	[Ag_2_L_2_]	AgC_6_H_6_NO_2_	244.0	57.4	34.4	2.45	5.73	34.2	2.46	5.02
**4**	[FeL_2_]	FeC_14_H_12_N_2_O_4_	328.1	36.6	51.2	3.66	8.53	49.8	3.98	7.89
**5**	[CoL_2_]	CoC_14_H_12_N_2_O_4_	331.2	37.2	50.7	3.62	8.45	50.4	3.81	7.87
**6**	[CuL_2_]	CuC_14_H_12_N_2_O_4_	335.8	38.1	50.0	3.57	8.33	49.7	3.70	7.75
**7**	[MnL_2_]	MnC_14_H_12_N_2_O_4_	327.2	36.5	51.3	3.67	8.56	51.1	3.74	7.91
**8**	[AlL_3_].3H_2_O	AlC_21_H_21_N_3_O_9_	489.0	35.0	51.5	4.90	8.59	51.8	4.43	8.05
**9**	[NiL_2_]	NiC_14_H_12_N_2_O_4_	331.0	37.1	50.8	3.63	8.46	51.4	3.84	7.94
**10**	[CrL_2_. 4H_2_O]. NO_3_	Cr C_14_H_20_N_2_O_8_	456.3	49.6	36.8	4.38	9.20	37.0	4.85	14.0

**Fig. 2:**
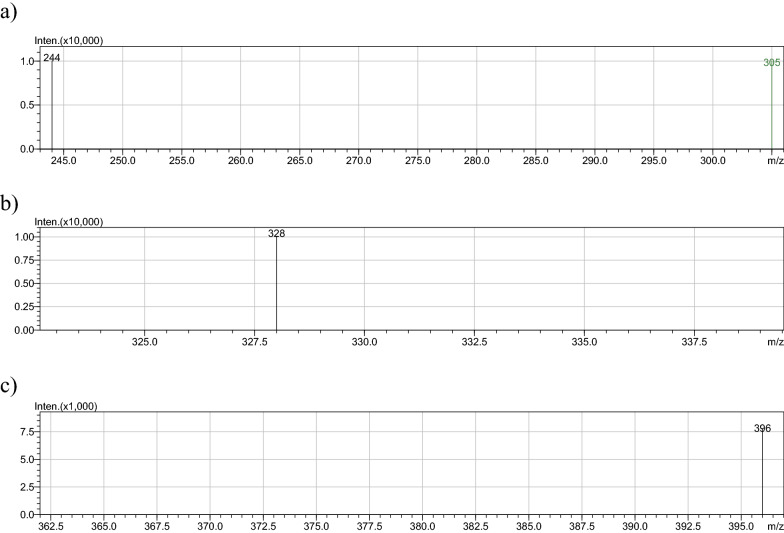
Electrospray Ionization Mass spectra of anthranilic acid metal complexes of **a** Ag (I) and **b** Cr (III) **c** Fe (II)

Electrospray ionization mass spectrometry (ESI–MS) was used to characterize three complexes namely Ag (I) complex **3**, Fe (II) complex **4** and Cr (III) complex **10**. The spectra are given in Fig. 2. ESI is ideal for the mass determination of the metal complexes since it provides information on the parent ion, with little or no fragmentation. It also provides data on the complex in the cationic form (by running in negative polarity) and affords clues to the presence of counter ions. Thus, the neutral complexes **3** and **4** showed M^+.^ peaks at 244 and 328 Da respectively, whereas Cr (III) complex **10**, showed a peak at 396 Da which corresponds to the [CrL_2_(H_2_O)_4_] ^+^ confirming the presence of NO_3_^−^.

**Fig. 3:**
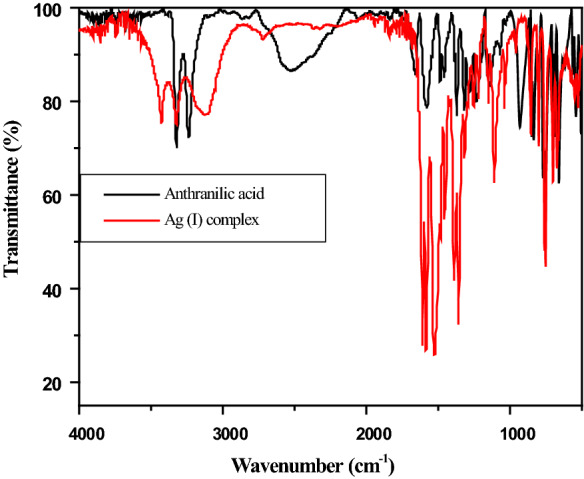
FTIR Spectra of anthranilic acid and Ag (I) complex **3**

FTIR spectra of anthranilic acid metal complexes **(1–10)** were also recorded and are presented in the Fig. 3. All metal complexes display two well-defined peaks  asymmetric stretches of the amino (–NH_2_) group at ~ 3300 cm^−1^ and ~ 1600 cm^−1^ due to carboxylic (–COO) group respectively. The OH stretch of the carboxylic group does not appear in the IR spectra of the complexes because they were deprotonated during the synthesis with aqueous sodium hydroxide. The IR data is summarized in Table 1. The carboxyl stretches of complexes (**1–10**) show major shift from that of native anthranilic acid, reflecting both the deprotonation as well coordination to the metal centers.

**Fig. 4:**
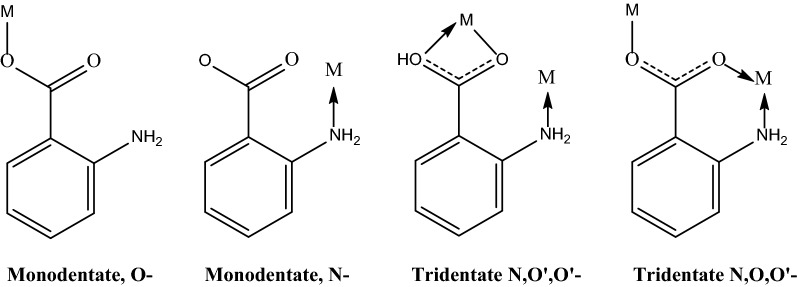
Modes of coordination between anthranilic acid and some metals

Given that the *ortho* disposition of the amino and carboxyl groups of anthranilic acid, there are several possibilities of chelate formation [38]. X-ray studies obtained from the Cambridge Structural Database (CSD) of some these complexes show monodentate (Ag_2_L_2_), tridentate N,O,O′ type (MnL_2_, CuL_2_, ZnL_2_) and tridentate N,O′,O′ type (BiL_3_) types of coordination (Fig. 4).

**Fig. 5:**
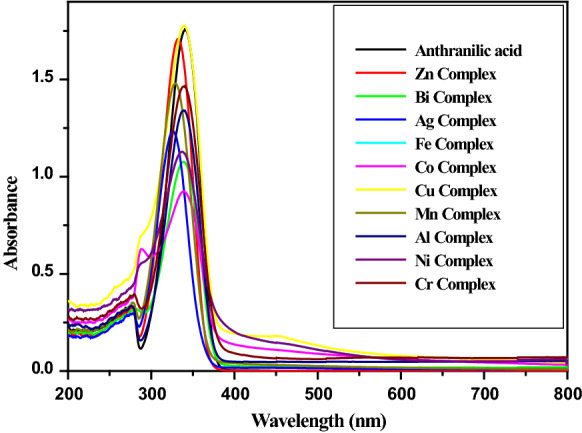
UV–visible spectra of the anthranilic acid and the complexes (**1–10**)

The UV–visible spectra of the anthranilic acid and complexes **1–10** were recorded in DMSO. The complexes were insoluble or sparingly soluble in water and ethanol. The absorption peak of anthranilic acid at 340.5 nm, attributed to π → π* transition, is blue shifted upon complexation with metal ions (Fig. 5). The shift ranged from 0.5 nm ([CrL_2_.4H_2_O].NO_3_, **10**) and 14.5 nm (Ag_2_L_2_, **3**).

Molar magnetic susceptibility (Χ_m_) data were used to obtain the magnetic moment (µ, in Bohr magnetons) of the complexes, following literature procedure, and using the following equations:

1 $${\text{X}}_{{\text{m}}} = {\text{X}}_{{\text{g}}}* \,{\text{F}}.{\text{W}}$$

2 $$\mu = 2.84\sqrt {\left( {{\text{Xm}}*\,{\text{T}}} \right)}$$

where X_g_ is measured mass magnetic susceptibility, F.W is the formula weight of the complex, and T is the temperature in Kelvin.

Zn, Bi, Ag and Al metal complexes are diamagnetic, whilst the remaining metal complexes are paramagnetic with some being high spin complexes such as those containing Fe(II) **4**, Co(II) **5**, Mn(II) **7**, and Cr(III) **10**.

The spin-only magnetic moments for high spin Fe(II) and Co(II) are 4.90 and 3.87 B.M, compared to the experimental values of 5.1 and 4.6 B.M. respectively. The higher experimental values can be attributed to orbital contributions and spin–orbit coupling contributions to the total magnetic moment. Typical magnetic moment values for tetrahedral complexes of Fe(II) lie in the range 5.0–5.6 B.M, and for tetrahedral Co(II) in the range 4.2–5.3 B.M. Octahedral complexes typically exhibit higher orbital contributions to the magnetic moment, resulting in ranges of 5.1–5.7 B.M. for Fe(II) and 4.3–5.2 B.M. for Co(II). The experimentally determined magnetic moments are consistent with either geometry, but the 1:2 stoichiometry is consistent with the tetrahedral structures only.

**Fig. 6:**
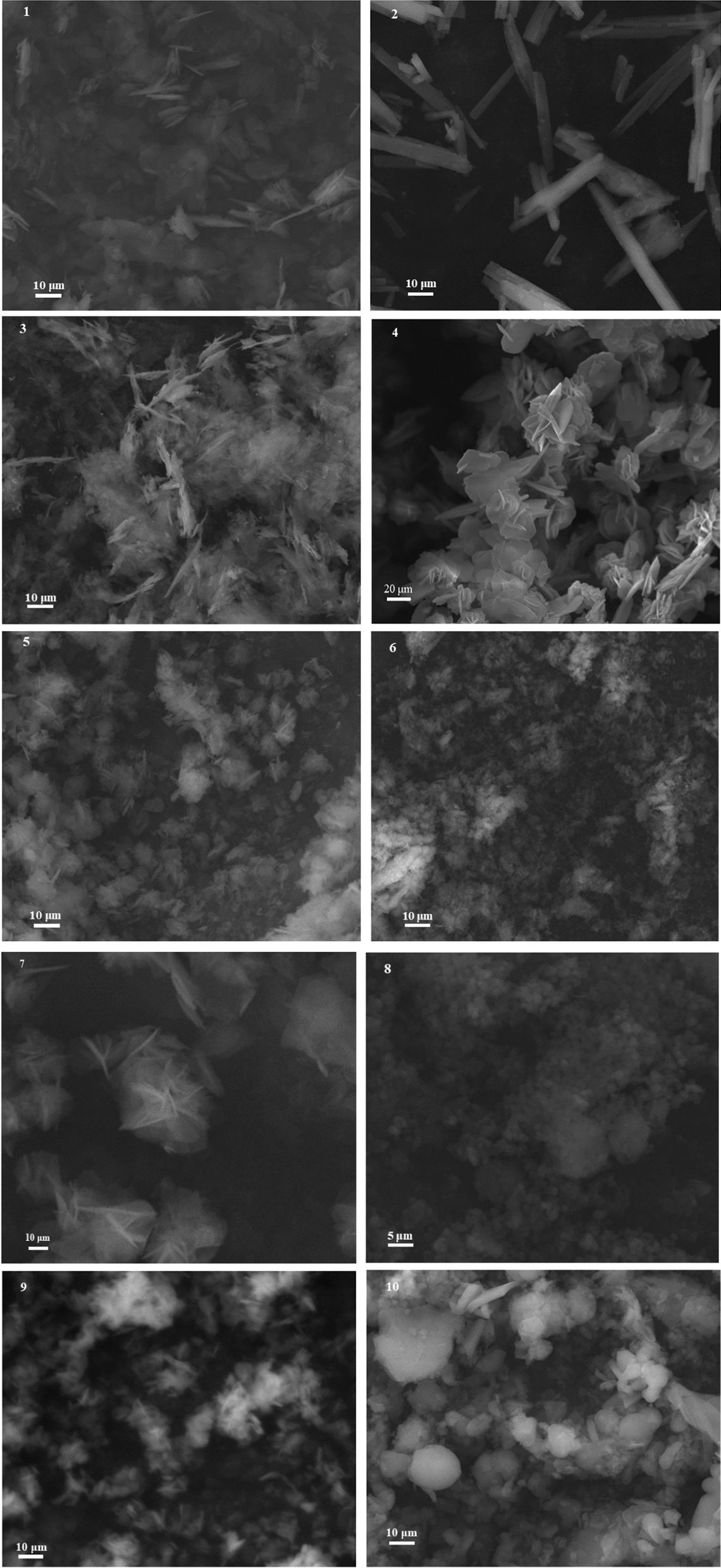
SEM images of anthranilic acid metal complexes (**1–10**)

Figure 6 shows SEM images of anthranilic acid metal complexes (**1–10**). It was noticed that metal complexes (**1–10**) showed different morphology and size. Metal complexes **1, 3** and **4** were plate-like, metal complex **2** was in the form of nanorods and metal complexes **8** and **10** showed sphere-like morphology. Semi-flower morphology was observed in the case of metal complexes **5, 7** and **9** while metal complex **6** was amorphous.

**Fig. 7:**
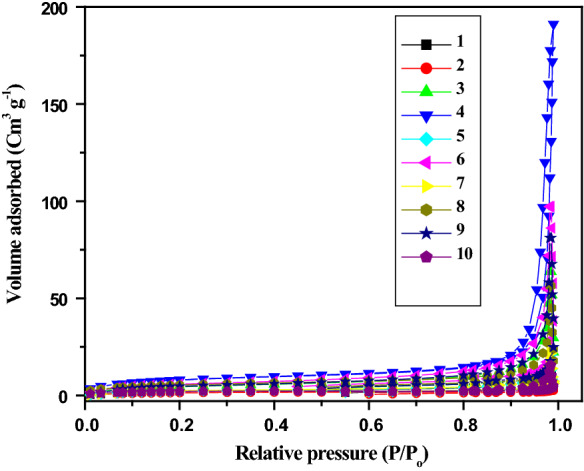
N_2_-adsorption–desorption isotherms of anthranilic acid metal complexes (**1–10**)

The surface area and porosity of anthranilic acid metal complexes (**1–10**) were determined by N_2_-adsorption–desorption analysis. Figure 7 displays the N_2_-adsorption–desorption isotherms and pore size distribution of anthranilic acid metal complexes (**1–10**). The shape of the isotherms reveals typical type-IV curve having narrow H3-type hysteresis loop, demonstrating the existence of mesoporous particles.

The BET surface area of metal complexes (**1–10**) along with pore size and pore volume are shown in Table 3. The BET surface area of metal complex **4** was higher (27.41 m^2^ g^−1^) than other metal complexes followed by complex **6** with surface are 20.27 m^2^ g^−1^. The surface area of complexes **3, 8** and **9** was ranged 16.28–18.30 m^2^ g^−1^. Metal complexes **1, 5, 7** and **10** revealed surfaces area between 7.08 and 8.53 m^2^ g^−1^. The metal complex **2** showed lowest surface area, 4.88 m^2^ g^−1^ (Table 3).

**Table 3:** BET surface area and texture properties of metal complexes (**1–10**)

Compounds	S_BET_ (m^2^g^−1^)	Dp (nm)^a^	Vp (cm^3^ g^−1^)^b^
1	7.72	17.27	0.0289
2	4.88	17.59	0.0123
3	16.28	22.56	0.0980
4	27.40	43.88	0.2951
5	8.16	15.67	0.0319
6	20.27	28.34	0.1499
7	8.53	18.83	0.0329
8	18.30	18.42	0.0884
9	17.05	26.74	0.1248
10	7.08	16.50	0.0237

^a^Dp (pore size) was estimated from BJH desorption determination

^b^Vp (pore volume) was determined using the adsorption branch of the N_2_ isotherm curve at *P/P*_*0*_ = 0.99

### DFT study

**Fig. 8:**
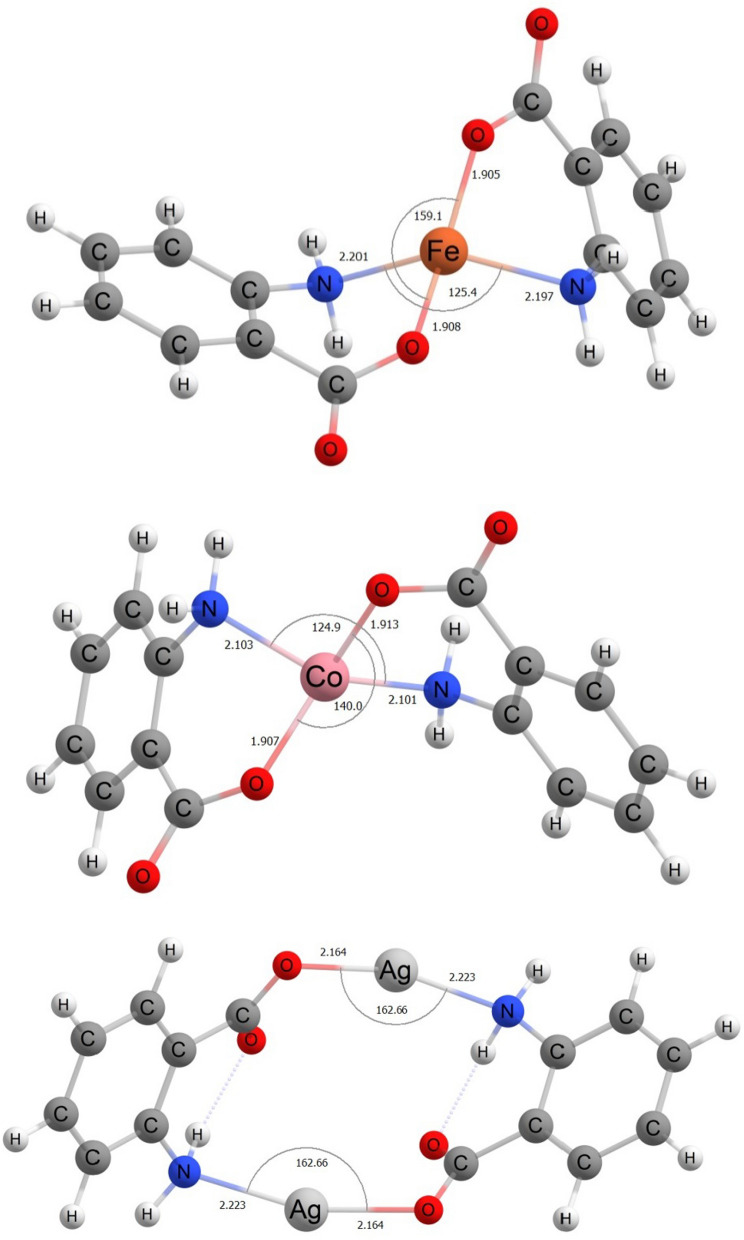
Optimized structures of Fe(II), Co(II) and Ag(I) complexes

DFT calculations were performed on Co(II), Fe(II) and Ag(I) complexes. The choice of these complexes was based on the antipathogenic and catalytic activities (Co complex **5** and Ag complex **3**) and their lack thereof (Fe complex **4**). The optimized Ag(I) adopts a near-linear coordination geometry with O-Ag–N angles of 162.7°. The lowest energy structure found exhibits C2 molecular symmetry (Fig. 8). Co(II) and Fe(II) adopt distorted tetrahedral geometries with the geometry of Fe(II) approaching disphenoidal with an O–Fe–O angle of 159.1° (Fig. 8). High-spin quintet and quartet states for Fe(II) and Co(II) respectively were determined to correspond to the ground states, consistent with the experimentally determined magnetic moments.

**Fig. 9:**
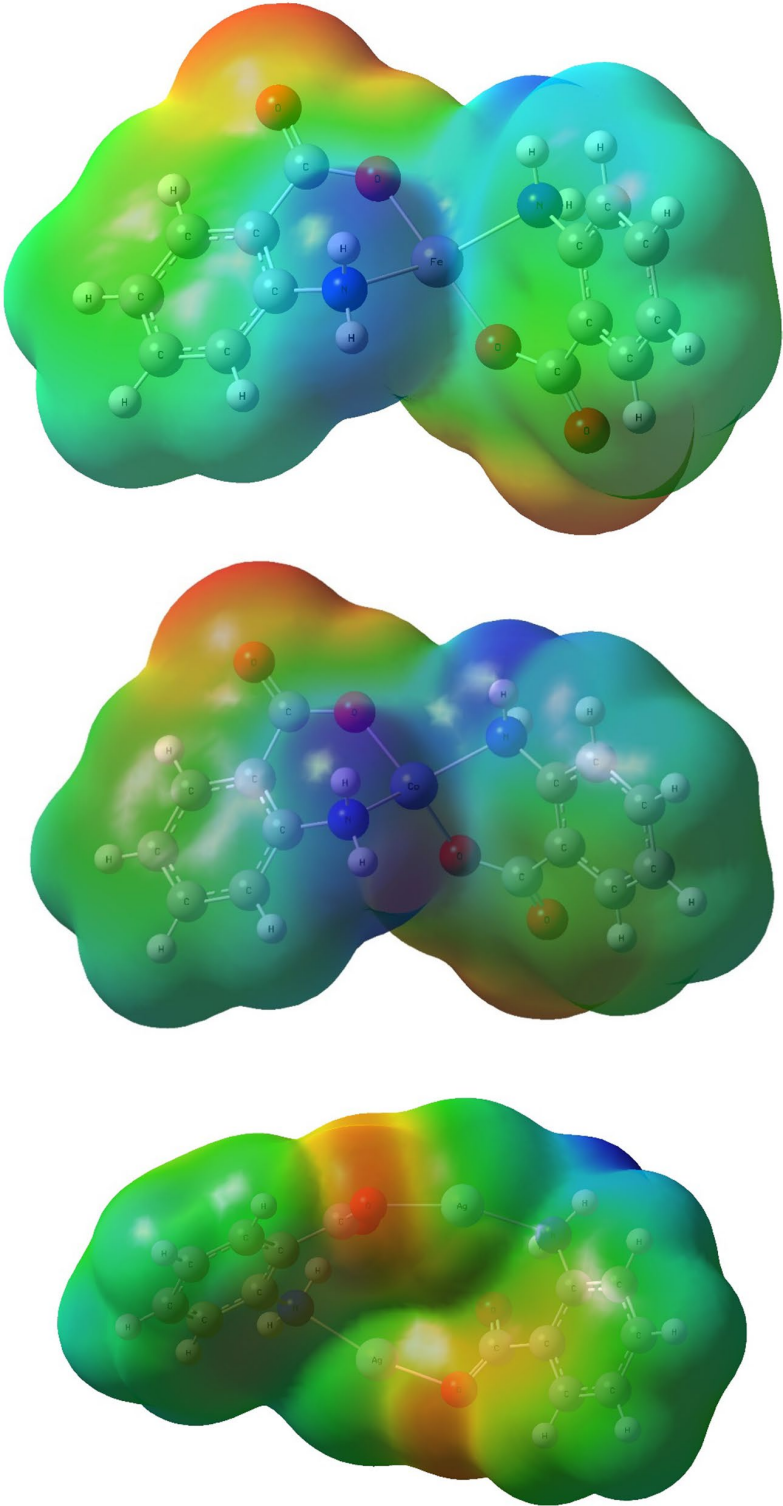
Molecular electrostatic potential surface maps of Fe(II), Co(II) and Ag(I) complexes

Molecular electrostatic potential surface maps were also determined to provide three-dimensional charge distributions of complexes. Besides providing invaluable information on the shape of the complexes, these charge distributions also give an insight in the types and strength of interaction between the complexes and other molecules. Ag_2_L_2_ complex **3** shows an almost neutral distribution of charges, whereas FeL_2_ complex **4** and CoL_2_ complex **5** shows pronounced positive centers around the metal ion core, with moderate negative charge distribution around the carboxyl group (Fig. 9).

### Catalytic activity

**Fig. 10:**
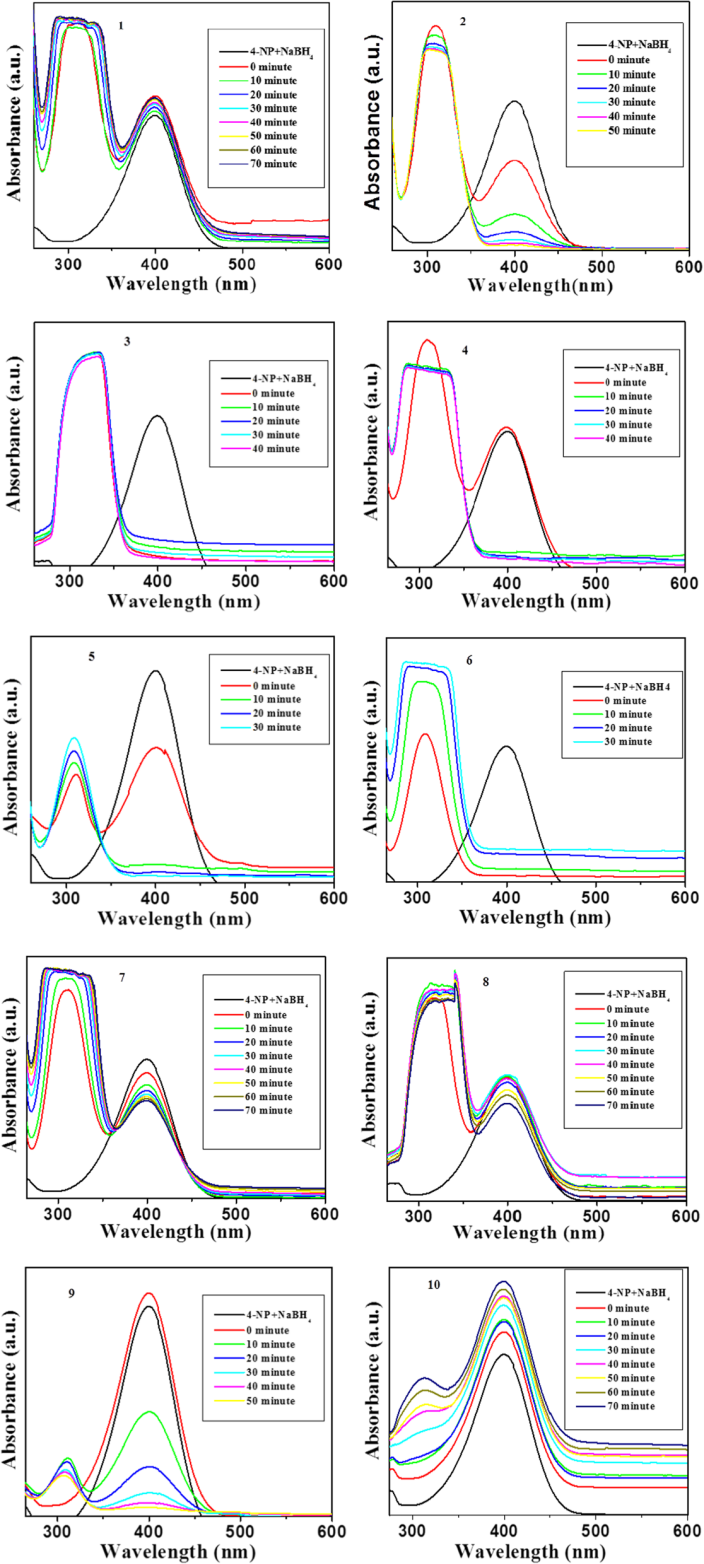
Spectral variation of 4-nitrophenol over anthranilic acid metal complexes (**1–10**)

**Fig. 11:**
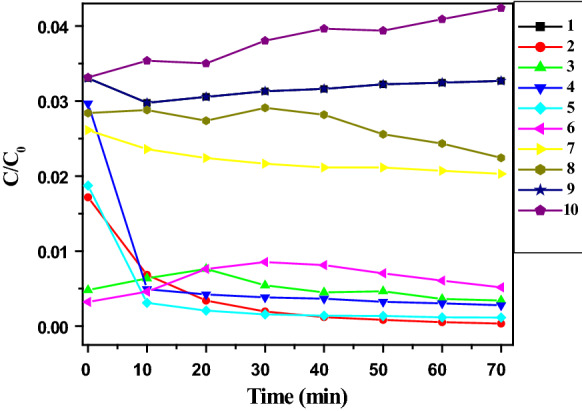
Catalytic conversion of 4-nitrophenol over anthranilic acid metal complexes (**1–10**)

The catalytic activity of anthranilic acid metal complexes (**1**–**10**) was studied for the reduction of 4-NP at room temperature. Figure 10 displays the UV–visible absorption spectra of 4-NP at different times in the presence of anthranilic acid metal complexes (**1**–**10**). The progressive attenuation, with time, of the absorption peak of 4-NP at 400 nm and the appearance of a new peak at 300 nm, signal the formation of 4-aminophenol (4-AP). However, there was no reduction of 4-NP without catalysts (Fig. 10). The peak observed at 400 nm was due to the formation of phenolate ion in the presence of NaBH_4_ [20, 21] and reaction was initiated in the presence of anthranilic acid metal complexes (**1**–**10**) only. Although, thermodynamically, the reduction of 4-NP by NaBH_4_ is feasible due to high potential of NaBH_4_ (− 1.33 V) as compared with 4-NP (− 0.76 V) the reaction is not initiated in the presence of NaBH_4_ alone, indicating that the reaction is kinetically controlled [39–41]. Thus, a catalyst is necessary for the reduction of 4-NP. Figure 11 shows the variation of concentration of 4-NP as a function of reaction time in the presence of anthranilic acid metal complexes (**1**–**10**), where C is the remaining concentration and C_o_ is the initial concentration of 4-NP. It is worth noticing that different anthranilic acid metal complexes (**1**–**10**), showed different activity. The metal complexes **5** and **6** revealed highest activity for the reduction of 4-NP and conversion was accomplished within 30 min. Metal complexes **2** and **9** showed low activity as compare with complexes **5, 6, 3** and **4** while reduction of 4-NP was achieved within 50 min. However, other anthranilic acid metal complexes such as **1, 7, 8**, and **10** did not show catalytic activity even after 70 min.

### Anti-bacterial, anti-fungal and nematocidal activities

Anti-bacterial potential of the synthesized anthranilic acid metal complexes (**1**–**10**), was assessed against clinically significant strains of *S. aureus*, *P. aeroginosa* and *E. coli*. The metal complexes exhibited varied response against different strains. However, metal complex **3** demonstrated good antibacterial activity against all tested bacterial strains at two tested concentrations. Larger zones of inhibition were observed at higher concentrations (1000 ppm) compared to lower concentration (500 ppm). Metal complex **6** revealed antibacterial activity against *S. aureus* and *P. aeroginosa* at higher concentration (1000 ppm), however, remained ineffective against *E. coli* at both concentrations (Table 4). Most of the tested metal complexes showed no antifungal activities against *F. solani* and *A. niger* with the exception of complex **3** which had apparent antifungal activity against *F. solani* at higher concentrations. Metal complex **1** showed slight zone of inhibition against fast growing *A. niger*. Nematocidal activities of tested compounds were recorded at both concentrations at 96 h of exposure to J_2_ of *M. javanica*. Metal complex **3** showed 100% mortality of J_2_ of *M. javanica* at 24 h of exposure at both tested concentrations (500 and 1000 ppm). Other tested anthranilic acid metal complexes were found ineffective against J_2_ of *M. javanica*.

**Table 4:** Anti-bacterial, anti-fungal and nematocidal activities of metal complexes (**1–10**)

Complexes	Conc. (ppm)	*S. aureus*	*P. aeroginosa*	*E. coli*	*F. Solani*	*A. niger*	*M. javanica*
		Zone of inhibition (cm)					Mortality % at 96 h
**1**	1000	–	–	–	–	–	4.8 ± 1.7
	500	–	–	–	–	–	–
**2**	1000	–	0.67 ± 0.34	–	–	–	5 ± 1.6
	500	–	–	–	–	–	–
**3**	1000	1.63 ± 0.09	1.5 ± 0.058	1.27 ± 0.03	1.37 ± 0.15	0 ± 0	100 ± 0
	500	1.4 ± 0.11	1.03 ± 0.09	1.17 ± 0.03	–	–	100 ± 0
**4**	1000	–	–	–	–	–	–
	500	–	–	–	–	–	–
**5**	1000	0.8 ± 0.06	–	–	–	–	5.1 ± 1.0
	500	–	–	–	–	–	–
**6**	1000	0.73 ± 0.37	1.03 ± 0.03	–	–	–	6.7 ± 2.1
	500	–	–	–	–	–	5.3 ± 2.2
**7**	1000	0.8 ± 0.40	–	–	–	–	6.9 ± 2.2
	500	0.9 ± 0.21	–	–	–	–	3.4 ± 1.1
**8**	1000	–	1.33 ± 0.24	–	–	–	4.8 ± 1.3
	500	–	0.467 ± 0.24	–	–	–	–
**9**	1000	0.43 ± 0.22	–	–	–	0.6 ± 0	5.2 ± 1.6
	500	–	–	–	–	0.2 ± 0	–
**10**	1000	0.4 ± 0.21	–	–	–	–	4.7 ± 1.2
	500	–	–	–	–	–	–
Control+	With DMSO	–	–	–	–	–	7.5 ± 1.0
Control−	Without DMSO	–	–	–	–	–	–

A literature survey showed that the biological activities of metal complexes of anthranilic acid are not well studied. However, the activities of metal complexes of substituted anthranilic acids (e.g., mesalazine), N-aryl anthranilic acid derivatives (e.g., mefenamic, flufenamic and tolfenamic acids) [38] and imines of anthranilic acid [42] have been studied. The mesalazine complexes of Cr, Mn, Co, Ni, Cu and Zn showed antifungal and antibacterial activity. The bismuth complexes with mefenamic, flufenamic and tolfenamic acids showed antibacterial activity against *Helicobacter pylori*. However, these studies cannot be compared to the present work, as the bacteria and fungi are different. Furthermore, there are no reports of nematocidal studies of anthranilic acid and its derivatives.

## Conclusion

A series of nano-anthranilic acid metal complexes (**1**–**10**) with metals such as Zn(II), Bi(III), Fe(II), Co(II), Cu(II), Mn(II), Al(III), Ni(II), and Cr(III) were synthesized and characterized. TGA and CHN analysis data revealed that the stoichiometry of complexes was 1:2 metal/ligand except for Ag(I) (ratio 1:1), and Al and Bi (ratio 1:3). The structure of complexes **3**, **4** and **5** were optimized by DFT calculations and the charge distribution was also calculated. The Co(II) **5** and Cu(II) **6** metal complexes revealed higher activity for the reduction of 4-NP. Metal complex Ag(I) **3** showed good antibacterial, antifungal and nematocidal activities.

## Data Availability

All data generated or analyzed during this study are included in this published article.
